# *Gpr63* is a modifier of microcephaly in *Ttc21b* mouse mutants

**DOI:** 10.1371/journal.pgen.1008467

**Published:** 2019-11-15

**Authors:** John Snedeker, William J. Gibbons, David F. Paulding, Zakia Abdelhamed, Daniel R. Prows, Rolf W. Stottmann

**Affiliations:** 1 Division of Human Genetics, Cincinnati Children’s Hospital Medical Center, Cincinnati, Ohio, United States of America; 2 Neuroscience Graduate Program, University of Cincinnati College of Medicine, Cincinnati, Ohio, United States of America; 3 Department of Anatomy and Embryology, Faculty of Medicine (Girl’s Section), Al-Azhar University, Cairo, Egypt; 4 Department of Pediatrics, University of Cincinnati College of Medicine, Cincinnati, Ohio, United States of America; 5 Division of Developmental Biology, Cincinnati Children’s Hospital Medical Center, Cincinnati, Ohio, United States of America; 6 Shriner’s Hospital for Children - Cincinnati, Cincinnati, Ohio, United States of America; The Jackson Laboratory, UNITED STATES

## Abstract

The primary cilium is a signaling center critical for proper embryonic development. Previous studies have demonstrated that mice lacking *Ttc21b* have impaired retrograde trafficking within the cilium and multiple organogenesis phenotypes, including microcephaly. Interestingly, the severity of the microcephaly in *Ttc21b*^*aln/aln*^ homozygous null mutants is considerably affected by the genetic background and mutants on an FVB/NJ (FVB) background develop a forebrain significantly smaller than mutants on a C57BL/6J (B6) background. We performed a Quantitative Trait Locus (QTL) analysis to identify potential genetic modifiers and identified two regions linked to differential forebrain size: *modifier of alien QTL1 (Moaq1)* on chromosome 4 at 27.8 Mb and *Moaq2* on chromosome 6 at 93.6 Mb. These QTLs were validated by constructing congenic strains. Further analysis of *Moaq1* identified an orphan G-protein coupled receptor (GPCR), *Gpr63*, as a candidate gene. We identified a SNP that is polymorphic between the FVB and B6 strains in *Gpr63* and creates a missense mutation predicted to be deleterious in the FVB protein. We used CRISPR-Cas9 genome editing to create two lines of FVB congenic mice: one with the B6 sequence of *Gpr63* and the other with a deletion allele leading to a truncation of the GPR63 C-terminal tail. We then demonstrated that *Gpr63* can localize to the cilium *in vitro*. These alleles affect ciliary localization of GPR63 *in vitro* and genetically interact with *Ttc21b*^*aln/aln*^ as *Gpr63;Ttc21b* double mutants show unique phenotypes including spina bifida aperta and earlier embryonic lethality. This validated *Gpr63* as a modifier of multiple *Ttc21b* neural phenotypes and strongly supports *Gpr63* as a causal gene (i.e., a quantitative trait gene, QTG) within the *Moaq1* QTL.

## Author summary

*TTC21B* in humans is a known ciliopathy gene and contributes to the pathophysiology of a number of ciliopathies. Mice homozygous for a null allele of *Ttc21b* also have a spectrum of ciliopathy phenotypes, including microcephaly (small brain). Further work has shown that the severity of the microcephaly significantly depends on the genetic background of the mouse model. The genetic mechanisms of the *Ttc21b* pathophysiology and the interacting gene network remain far from understood. As an initial attempt to understand the underlying mechanism(s) underlying the variable effects on brain size, we performed a quantitative trait locus (QTL) analysis and found two regions of genomic significance that correlated with smaller brain size. We confirmed both QTLs with congenic lines. One of the two regions was small enough that we considered candidate genes and hypothesized *Gpr63* might be a contributing locus for a number of reasons. We evaluated this hypothesis directly with precise variant creation using genome editing and provide evidence that *Ttc21b* and *Gpr63* do indeed genetically interact. Thus, we have been able to combine classical QTL analysis and genome editing to directly test the resulting hypothesis.

## Introduction

Primary cilia are microtubule-based organelles known to play essential roles in proper development and function of a number of organ systems including the central nervous system (CNS) [1–4]. Ciliopathies are a class of human diseases caused by pathogenic variants in genes encoding proteins responsible for the proper form and function of cilia [5–7]. The frequent presentation of cognitive impairment in ciliopathy patients, in addition to severely compromised brain development in a number of ciliary mutant mouse models, clearly displays the importance of primary cilia in CNS health and development [8, 9]. Primary cilia have been implicated in transducing and regulating several critical developmental pathways including SHH, WNT, PDGF, TGFβ/BMP, RTK, and Notch [3, 10]. An array of cell surface receptors, including G-protein coupled receptors (GPCRs), localize to the ciliary membrane to modulate these pathways and other signaling events [11]. Intraflagellar transport (IFT) proteins are responsible for the movement of cargo within the cilium with complex-B proteins regulating anterograde transport from the basal body to the distal tip of the cilium and complex-A proteins regulating retrograde transport [12]. IFT-A proteins have also been shown to specifically play a role in the trafficking of select GPCRs in and out of the cilium [13–15].

*Tetratricopeptide repeat domain 21b (Ttc21b; Thm1; Ift139*; MGI 1920918) encodes an IFT-A protein necessary for the proper rate of retrograde trafficking within the primary cilium [16]. TTC21B is not part of the “core IFT-A complex” [15]. When TTC21B is reduced, a large portion of the remaining IFT-A protein subunits remains intact as this “core” complex and continues to interact with the dynein motor powering retrograde movement. Thus, TTC21B likely acts to facilitate binding of peripheral cargo proteins to the core proteins of the complex, thus assisting the transport of these cargo proteins through the cilium. Proper ciliary trafficking is known to be critical for the processing of GLI transcription factors, which are in turn essential for proper regulation of the SHH pathway [17, 18]. Mutant mice homozygous for the *alien* (*aln*) null allele of *Ttc21b* display impaired processing of GLI3, the primary repressor of SHH target genes, and consequently show an increase in SHH pathway activity in multiple tissues including the developing forebrain [16, 19].

The ciliopathies are only one example of a class of human diseases with broad phenotypic variability. The advent of genome sequencing is facilitating the study of genes and variants affecting disease penetrance and expressivity. The ability to specifically model such modifiers in experimental organisms like mice with genome editing should allow more in-depth studies of genetic interactions and their role in human disease. It is well known that genetic backgrounds of mouse inbred strains can affect phenotypic severity and penetrance of specific mouse mutations. These differences can be used to detect genetic interactions in complex traits and begin to understand the mechanisms leading to this variability [20, 21]. One example of genetic background influencing the expressivity of a phenotype is the forebrain malformations seen in *Ttc21b*^*aln/aln*^ mutants. Here we present a QTL analysis to further understand the underlying pathogenic molecular mechanism leading to the microcephaly. We demonstrate that *Gpr63* interacts with *Ttc21b* to modify the size of the forebrain in *Ttc21b*^*aln/aln*^ null mutants and provide evidence that the *Gpr63* mutation is the likely causal variant for the chromosome 4 QTL affecting brain size.

## Results

### *Ttc21b*^*aln*^ microcephaly is background dependent

**Fig 1:**
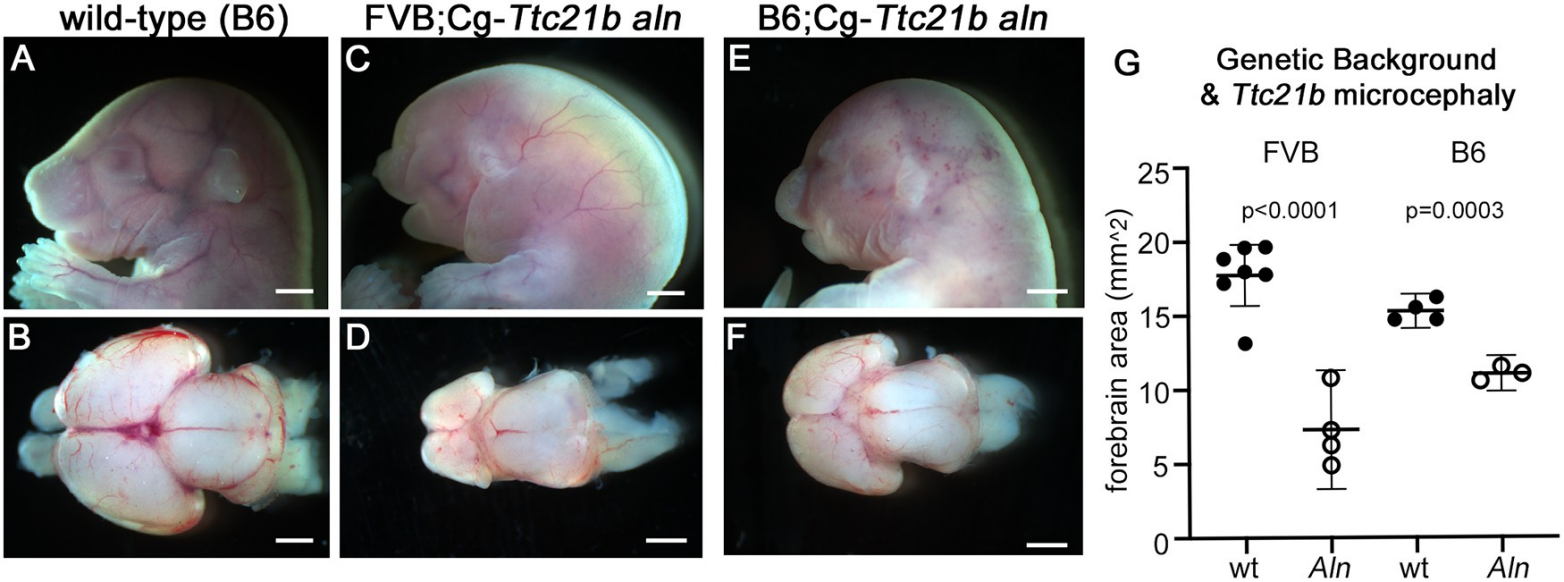
Genetic background affects the *Ttc21b*^*aln/aln*^ microcephaly phenotype. (A-F) Embryos and whole mount brains viewed from the dorsal aspect from control (A,B, E17.5), FVB;Cg-*Ttc21b*^*aln/aln*^ mutants (C,D, E18.5), and B6;Cg-*Ttc21b*^*aln/aln*^ mutants (E,F, E17.5). (G) Relative forebrain dorsal surface areas between wild-type and *Ttc21b*^*aln/aln*^ mutants from each genetic background. Data shown are mean ± 95% confidence interval. n = 7, 4, 4, 3 sample points. Scale bars in A-F indicate 1mm.

The phenotypes observed in *Ttc21b*^*aln/aln*^ mutants were first identified as part of an ENU mutagenesis experiment to identify recessive developmental phenotypes in the perinatal mouse [22]. A/J mice were treated with the ENU mutagen and the subsequent breeding and mapping of the causative mutation involved an outcross to the FVB/NJ (FVB) strain [16, 22]. *Ttc21b*^*aln/aln*^ homozygous mutants on this mixed A/J;FVB background exhibited multiple forebrain and craniofacial phenotypes (Fig 1A–1D) [16, 19]. While the microcephaly phenotype was completely penetrant, craniofacial phenotypes such as cleft lip and palate were only present in a minority of embryos. To increase the frequency of craniofacial phenotypes and facilitate future molecular analyses, the *Ttc21b*^*aln*^ allele was serially backcrossed onto the C57BL/6J (B6) strain. The B6 genetic background has previously been shown to be more susceptible to craniofacial phenotypes in a number of studies [23–26]. This initial, exploratory backcross was done informally and was not genotyped. After the *Ttc21b*^*aln*^ allele was serially backcrossed to the B6 background for at least five generations, we noticed the relative size of the forebrain tissues in B6.Cg-*Ttc21b*^*aln/aln*^ mutants increased substantially as compared to the mutants previously analyzed, although they remained smaller in all *Ttc21b*^*aln/aln*^ mutants than control brains and still lacked olfactory bulbs (Fig 1E and 1F). To confirm this was indeed an effect of varying the genetic background, the *Ttc21b*^*aln*^ allele was again serially backcrossed to the FVB background for at least three generations. Embryonic analysis showed the severity of microcephaly initially seen was recovered in the new FVB.Cg-*Ttc21b*^*aln/aln*^ mutants (Fig 1C and 1D). We measured the forebrain surface area of *Ttc21b*^*aln/aln*^ mutants on both FVB and B6 genetic backgrounds (Fig 1G) and saw a 60% decrease in forebrain size in FVB.Cg-*Ttc21b*^*aln/aln*^ animals as compared to littermate controls (n = 7 wt and n = 4 *aln* mutants; p<0.0001), but only a 30% decrease in B6.Cg-*Ttc21b*^*aln/aln*^ animals (n = 4 wt and n = 3 *aln* mutants; p = 0.00033). The interaction between genetic background and the *Ttc21b*^*aln*^ allele was analyzed with a 2-way ANOVA (p = 0.0053).

Given all of this preliminary data, we hypothesized the genetic background effect on *Ttc21b*^*aln/aln*^ mutant forebrain size would allow a Quantitative Trait Locus (QTL) analysis to generate insight into the underlying molecular mechanism(s). In order to generate mice as genetically uniform as practical prior to performing a QTL analysis, we further backcrossed both strains of *Ttc21b*^*aln*^ mice. After at least three further generations of backcrossing onto FVB (FVB;B6-*Ttc21b*^*aln/aln*^) and four generations onto B6 (B6;FVB-*Ttc21b*^*aln/aln*^), we performed a genome-wide high-density MegaMUGA SNP scan and confirmed that each strain was approximately 90% pure (S1 Table). We further purified the FVB and B6 advanced backcross lines with at least two additional backcrosses combined with targeted microsatellite marker screening of chromosomal areas not yet homozygous for FVB or B6 as identified in the initial genome scan. This allowed us to create FVB.Cg-*Ttc21b*^*aln/wt*^ animals that were >99% FVB and B6.Cg-*Ttc21b*^*aln/wt*^ animals purified to ~97% B6 background, as assayed by a GigaMUGA genome SNP scan providing strain-specific sequence information at over 143,000 SNPs [27] (S1 Table).

As we performed this breeding, we continued to note an incompletely penetrant exencephaly phenotype in *Ttc21b*^*aln/aln*^ animals, but the frequency did not appear to significantly differ between backgrounds (Table 1). We calculated the incidence of exencephaly and found that maintaining the *Ttc21b*^*aln*^ allele on either the FVB or B6 genetic backgrounds yielded exencephaly in ~40% of *Ttc21b*^*aln/aln*^ mutants (24/58, 41% in FVB;B6-*Ttc21b*^*aln/aln*^ and 30/65, 46% in B6;FVB-*Ttc21b*^*aln/aln*^). Note these embryos were collected for other experimental purposes as the incipient congenics were being further purified and are on a mixed genetic background.

**Table 1:** Mutant genotype and exencephaly incidence.

	**Total #**	***Ttc21b*** ^***aln/aln***^	**# expected**	**% with exencephaly**
aln.B6* **(E10.5-E18.5)**	193	42	48.25	46
aln.FVB* **(E10.5-E18.5)**	120	36	30	41
**aln.FVB.B6 F2 (E17.5)**	607	148	151.75	24
**aln.FVB (E17.5) incipient congenic (at >90% purity)**	86	22	21.5	95**
***Gpr63*.N2 (P28)**	35	8	8.75	0
	**Total #**	***Ttc21b*** ^***aln/aln***^; ***Gpr63* double mut**	**# expected**	**% with exencephaly**
***Gpr63***^***Ser(FVB)/Arg(B6)***^;***Ttc21b*** ^***aln/aln***^ **(E17.5)**	142	15	8.875	13
***Gpr63***^***Del/Arg(B6)***^;***Ttc21b*** ^***aln/aln***^ **(E17.5)**	142	13	8.875	46
***Gpr63***^***Del/Del***^;***Ttc21b*** ^***aln/aln***^ **(E17.5)**	128	8	8	25***
***Gpr63***^***Del/Del***^;***Ttc21b*** ^***aln/aln***^ (E12.5)****	72	9	5.375	89

* note these are from crosses before the QTL experiment was being rigorously conducted.

** exencephaly or earlier lethality

*** 6 of 8 were dead prior to ~E12.5 so no reliable exencephaly incidence measures

**** data are aggregated from *Gpr63*^*Del/wt*^;*Ttc21b* ^*aln/wt*^ intercrosses as well as *Gpr63*^*Del/Del*^;*Ttc21b* ^*aln/wt*^ x *Gpr63* ^*Del/wt*^;*Ttc21b* ^*aln/wt*^

We first generated F2 *Ttc21b*^*aln/aln*^ mutant progeny from a B6.FVB F1 (*Ttc21b*^*aln/wt*^*)* intercross for the QTL analysis. Fifty-four F2 litters were generated, which identified 148 *Ttc21b*^*aln/aln*^ mutant embryos from 607 total embryos. We excluded 35 mutants (24%) with exencephaly, which precluded a measurement of forebrain area. Ninety-six brains from the remaining F2 mutants were micro-dissected and the dorsal surface area of the forebrain was measured. Whole genome SNP genotyping of each F2 recombinant was carried out using a GigaMUGA panel and QTL mapping was performed using R/qtl [28] to identify chromosomal regions linked to differential brain sizes in *Ttc21b*^*aln/aln*^ mutants. The dataset used in the QTL analysis is presented in S2 Table and an overview of the QTLs identified and their effects on the phenotype is provided in Table 2.

**Table 2:** Summary of QTL effects.

Locus	LOD	VE (%)	↑ LOD	↑ VE (%)
*Moaq1* (Chr 4)	6.1	25.1	–	–
*Moaq2* (Chr 6)	4.7	20.1	–	–
*Ttc21b* (Chr2)	4.0	17.1	–	–
*Moaq1* + *2*	9.1	35.2	+ 3.05 *	+ 10.1
*Moaq1* + *Ttc21b*	9.9	37.4	+ 3.79 *	+ 12.3
*Moaq2* + *Ttc21b*	7.8	31.0	+ 3.09 *	+ 10.9
*Moaq1* + *2* + *Ttc21b*	12.4	44.4	+ 3.44 *^,^ ^#^	+ 9.9 ^#^

LOD scores and phenotypic variance explained (VE) were determined using R/qtl. ↑ LOD and ↑ VE represent the differences between the respective 1- and 2-loci models, or the 2-and 3-loci models.

* An ↑LOD score > +3 indicates a significant added contribution to the trait.

^#^ Difference in LOD score and VE in the 3-loci model is presented as the mean of the three separate comparisons with their respective 2-loci models.

**Fig 2:**
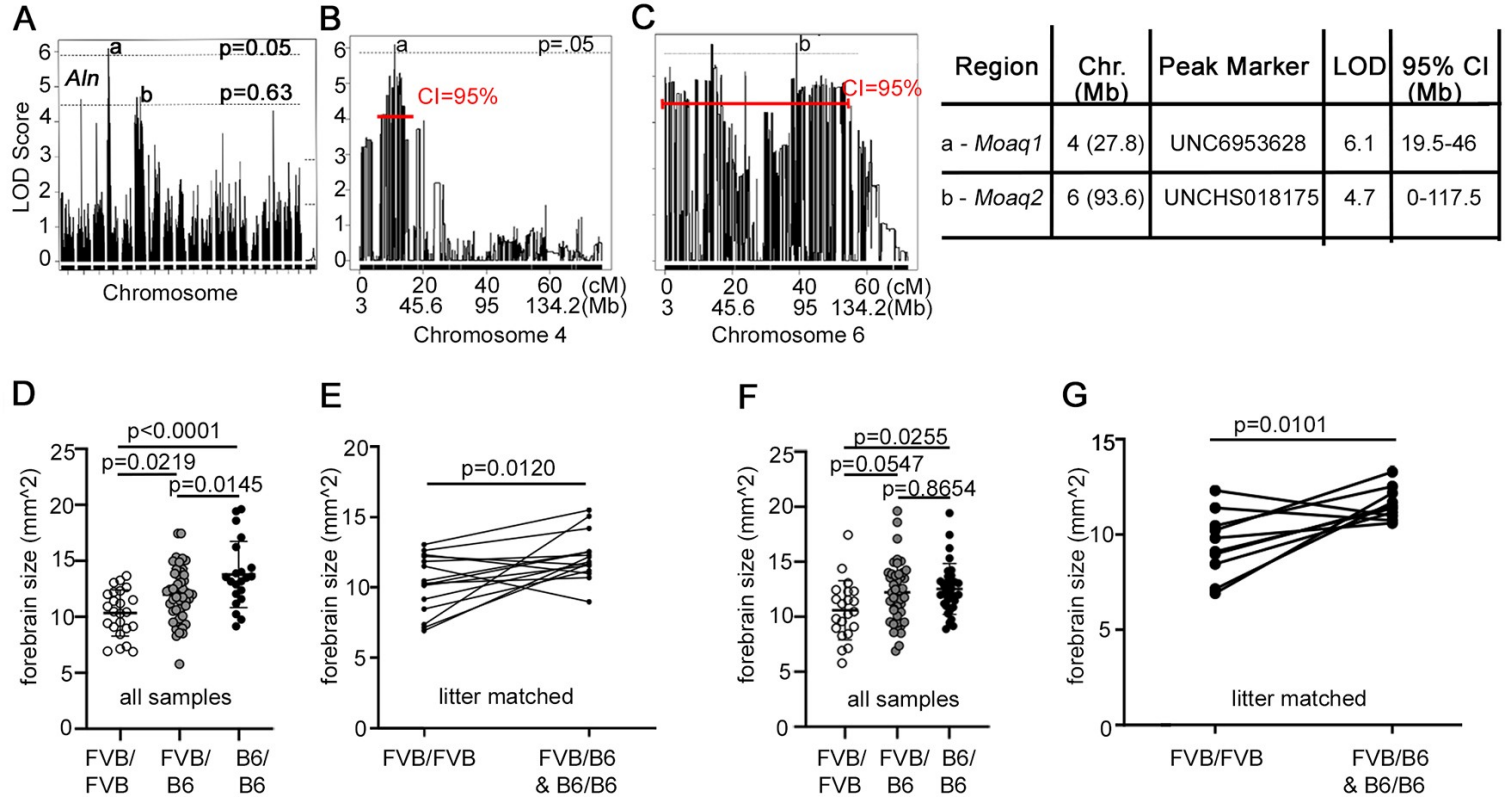
QTL analysis. (A) QTL LOD plots with dotted lines displaying genomewide significance scores of p = 0.05 and p = 0.63. (a) marks the significant QTL peak on chromosome 4, *Moaq1*, and (b) marks the suggestive *Moaq2* peak on chromosome 6. (B) Chromosome 4 QTL LOD plot and 95% confidence interval shown in red. (C) Chromosome 6 LOD plot and 95% confidence interval. (D) Analysis of brain size in *Ttc21b*^*aln/aln*^ homozygous for FVB genome identity, heterozygous for FVB/B6 and homozygous for B6 genome identity as genotyped by the peak marker for *Moaq1*. n = 24, 51, 21 sample points. (E) Analysis of brain size only among littermates in litters with both *Ttc21b*^*aln/aln*^*-Moaq1*^*FVB/FVB*^ and *Ttc21b*^*aln/aln*^*-Moaq1*^*FVB/B6 or B6/B6*^ embryos (n = 15 litters). (F) Analysis of brain size in animals homozygous for FVB genome identity, heterozygous for FVB/B6 and homozygous for B6 genome as genotyped by the peak marker for *Moaq2*. n = 24, 51, 21 sample points. (G) Analysis of brain size only among littermates in litters with both *Ttc21b*^*aln/aln*^*-Moaq2*^*FVB/FVB*^ and *Ttc21b*^*aln/aln*^*-Moaq2*^*FVB/B6 or B6/B6*^ embryos (n = 6 litters).

A significant QTL (genomewide p < 0.05; LOD 6.1) was mapped to chromosome 4 with its peak at 27.8 Mb (marker UNC6953268) and a 95% confidence interval spanning 19.5 Mb to 46 Mb (Fig 2A and 2B). This QTL explained 25.1% of the phenotypic variance (Table 2). We have named this chromosome 4 locus *modifier of alien QTL 1 (Moaq1)*. A second QTL was identified with a LOD score suggesting significance (genomewide p < 0.63; LOD 4.7) and mapped to chromosome 6 at 93.6 Mb (UNCHS018175) with a 95% confidence interval from 0 to 117.5 Mb (Fig 2A and 2C). This chromosome 6 QTL explained 20.1% of the trait variance and was named *modifier of alien QTL 2 (Moaq2)*. A third peak was found on chromosome 2 at the *Ttc21b* locus, which likely represents the A/J passenger background on which the initial mutagenesis was performed (Fig 2A). To assess the contribution of each locus to the trait, we determined the change in LOD score and percent variance explained for the three possible 2-loci models and the 3-loci model (Table 2). A significant increase in LOD score (>+3) was found for all comparisons, indicating that all three loci (*aln* and the 2 modifiers) significantly added to the phenotype, and together they explained 44.4% of the phenotypic variance. To assess whether the three loci interacted with each other and/or were additive, we performed a scantwo analysis in R/qtl (Table 2), using 1,000 permutations to establish the threshold levels for significance. Results indicated that none of the loci interacted (lod.int for all 3 pairings, not significant). However, the main effects of both *Moaq1* and *Moaq2* were independently additive with *aln*. The additive effect of *Moaq1* and *aln* was highly significant (lod.add = 9.13) and that for *Moaq2* and *aln* was significant (lod.add = 8.47). Interestingly, main effects of *Moaq1* and *Moaq2* were not additive (lod.add = 5.44; significance threshold = 8.29).

We confirmed a correlation between the genetic identity of *Moaq1* and *Moaq2* and an effect on forebrain size by comparing the genotype of the peak SNP marker for each QTL with forebrain size (Fig 2D and 2F). We first independently assessed F2 mice for genotype at the peak marker of the *Moaq1* locus and found an incremental decrease in brain size among *Ttc21b*^*aln/aln*^*-Moaq1*^*FVB/FVB*^ animals as compared to *Ttc21b*^*aln/aln*^*-Moaq1*^*FVB/B6*^ or *Ttc21b*^*aln/aln*^*-Moaq1*^*B6/B6*^ animals (Fig 2D). An overall ANOVA was performed to identify significant differences among these groups (p<0.0001) followed by a subsequent Tukey’s multiple comparison test analysis for comparison of each group (*Ttc21b*^*aln/aln*^*-Moaq2*^*FVB/FVB*^, *Ttc21b*^*aln/aln*^*-Moaq2*^*FVB/B6*^ and *Ttc21b*^*aln/aln*^*-Moaq2*^*B6/B6*^) with individual p-values ranging from 0.0145 to <0.0001 (Fig 2D). To control for a potential litter-based difference, we also compared only *Ttc21b*^*aln/aln*^*-Moaq1*^*FVB/FVB*^ mutants from litters with *Ttc21b*^*aln/aln*^*-Moaq1*^*FVB/B6*^ or *Ttc21b*^*aln/aln*^*-Moaq1*^*B6/B6*^ littermates (Fig 2E). Although this analysis necessarily used a smaller sample size than the non-litter matched comparison (Fig 2D), we again observed a 1.94mm^2^ reduction in *Ttc21b*^*aln/aln*^*-Moaq1*^*FVB/FVB*^ animals (p = 0.012).

A similar analysis testing just the *Moaq2* locus alone showed a significant variation among *Ttc21b*^*aln/aln*^*-Moaq2*^*FVB/FVB*^, *Ttc21b*^*aln/aln*^*-Moaq2*^*FVB/B6*^ and *Ttc21b*^*aln/aln*^*-Moaq2*^*B6/B6*^ animals (Fig 2F, ANOVA p = 0.0243). However, the subsequent analysis identified less striking differences between the groups (Fig 2F; p = 0.0255, 0.0547, 0.8654). We again complemented this with a matched littermate analysis and found *Ttc21b*^*aln/aln*^*-Moaq2*^*FVB/FVB*^ animals had approximately 20% smaller forebrains than littermates (Fig 2G; p = 0.0101).

### Congenic lines

**Fig 3:**
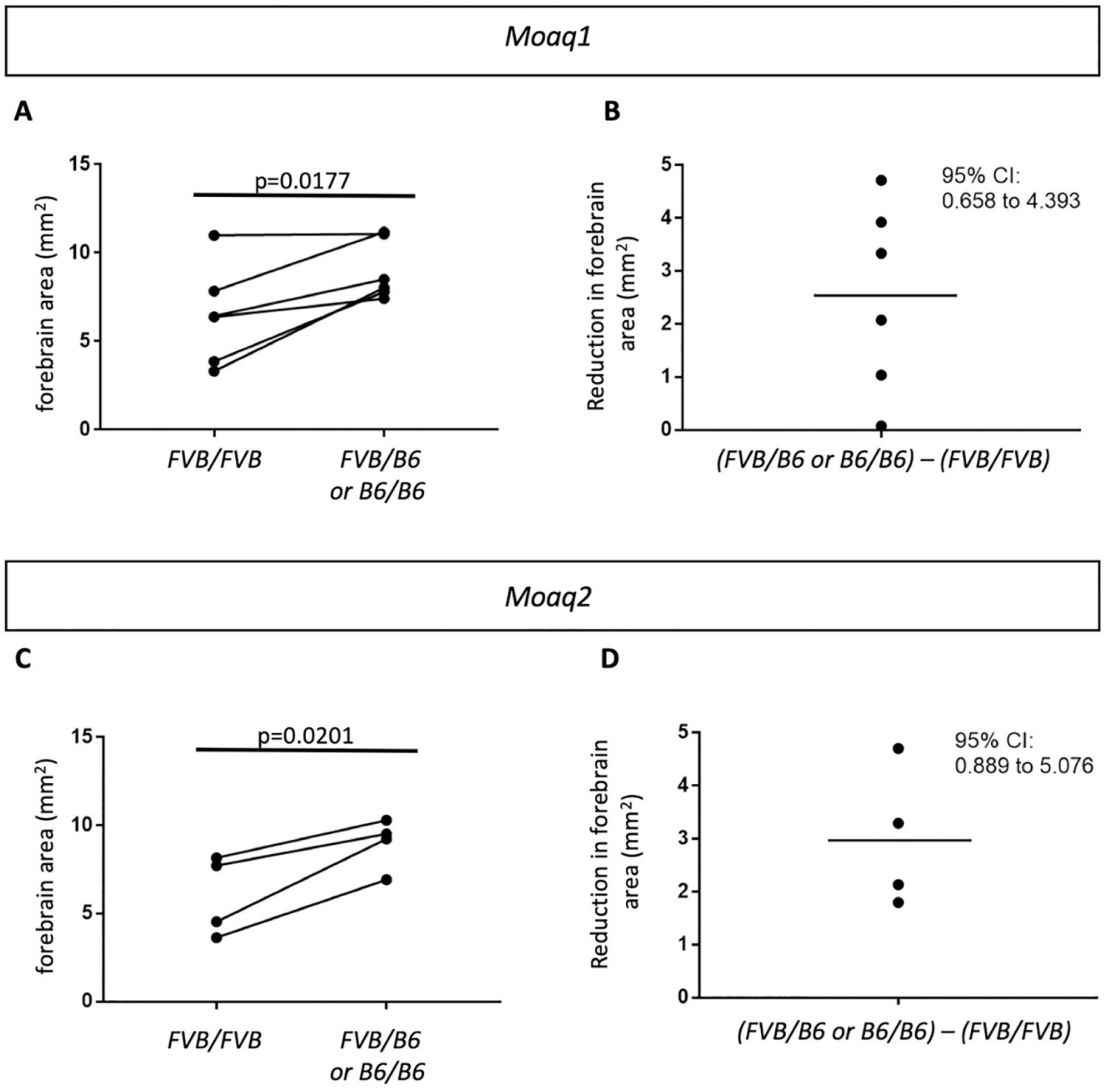
Congenic line validation of *Moaq1* and *Moaq2*. (A) Forebrain size from *Ttc21b*^*aln/aln*^*-Moaq1*^*FVB/FVB*^ (FVB/FVB) and *Ttc21b*^*aln/aln*^*-Moaq1*^*FVB/B6 or B6/B6*^ (FVB/B6 or B6/B6). (B) The amount of forebrain reduction in (FVB/B6 or B6/B6)–(FVB/FVB) brain sizes shows an average decrease of 2.24mm^2^. (C,D) Parallel analyses for *Moaq2* animals are shown.

We produced independent congenic lines to replicate the *Moaq1* and *Moaq2* loci. We noted in our original F2 population of 96 mice for the QTL analysis that no *Ttc21b*^*aln/aln*^ mutants were homozygous for B6 SNPs over a 10-Mb region on chromosome 14. We hypothesized this represented a region of the B6 genome that, in combination with homozygosity for the *Ttc21b*^*aln*^ allele, may lead to either early embryonic lethality and/or exencephaly. Thus, we attempted to avoid this complication and create FVB congenic lines with *Moaq1* and *Moaq2* genomic regions derived from the B6 strain with the hypothesis that the B6 genomic identity of *Moaq1* and *Moaq2* would increase the size of the *Ttc21b*^*aln/aln*^ over the size of the congenic FVB *Ttc21b*^*aln/aln*^ mutants. B6;FVB-*Ttc21b*^*aln/wt*^ F1 mice, (obligate heterozygotes across their genome), were repetitively backcrossed with FVB mice to produce a congenic strain that approached inbred FVB except for the heterozygous *Moaq1* or *Moaq2* B6 QTLs (*i*.*e*., FVB.Cg-*Moaq1*^*FVB/B6*^ and FVB.Cg-*Moaq2*^*FVB/B6*^). Mice from each congenic line were then intercrossed to determine if the B6-derived *Moaq1* and/or *Moaq2* QTLs could influence forebrain size on their own. During the process of creating these congenics, we noted that successive generations of mating onto the FVB background was leading to lethality of *Ttc21b*^*aln/aln*^ mutants prior to E17.5 or exencephaly. We observed *Ttc21b*^*aln/aln*^ mutants on a >99% FVB background and 21/22 either died prior to E17.5 or had exencephaly precluding measurement of brain size (Table 1). Note that while we had previously created mice that were largely enriched for FVB, even as much as 90%, (e.g., Fig 1), the incipient congenics created here for homozygote analysis are the purest FVB-*Ttc21b*^*aln/wt*^ population created to date. (While creating the mice used to perform the initial QTL analysis, we had not been intercrossing and dissecting embryos to create *Ttc21b*^*aln/aln*^ homozygotes so had not previously identified this effect on the phenotype.) This suggests a possibility for future work identifying a separate exencephaly modifier from this population. Given that the increasing incidence of exencephaly precluded an analysis of forebrain size in the congenic lines (N5 and greater), we analyzed animals from the earlier backcross generations. Littermate brains were analyzed using paired t-tests to account for potentially confounding variables, such as length of gestation and/or background differences based on the generation of backcross. The *Moaq1* QTL was found to affect brain size in FVB;B6-*Ttc21b*^*aln/aln*^ mutants in the N2 to N5 generations. *Ttc21b*^*aln/aln*^*-Moaq1*^*FVB/B6*^ and *Ttc21b*^*aln/aln*^*-Moaq1*^*B6/B6*^ animals had larger brains as compared to *Ttc21b*^*aln/aln*^*-Moaq1*^*FVB/FVB*^ (Fig 3A, p = 0.0177), with an average decrease of 2.24mm^2^ (Fig 3B). The *Moaq2* QTL was also validated using this strategy and *Ttc21b*^*aln/aln*^*-Moaq1*^*FVB/B6*^ and *Ttc21b*^*aln/aln*^*-Moaq1*^*B6/B6*^ animals again had larger brains than *Ttc21b*^*aln/aln*^*-Moaq1*^*FVB/FVB*^ (Fig 3C, p = 0.0201), with an average decrease of 3.40mm^2^ (Fig 3D). Thus, we conclude the QTL analysis of just 96 B6;FVB-*Ttc21b*^*aln/aln*^ F2 forebrains revealed two validated, novel QTLs that modify the effect of the *Ttc21b*^*aln/aln*^ mutation on forebrain size.

### Candidate genes for *Moaq1* QTL

**Fig 4:**
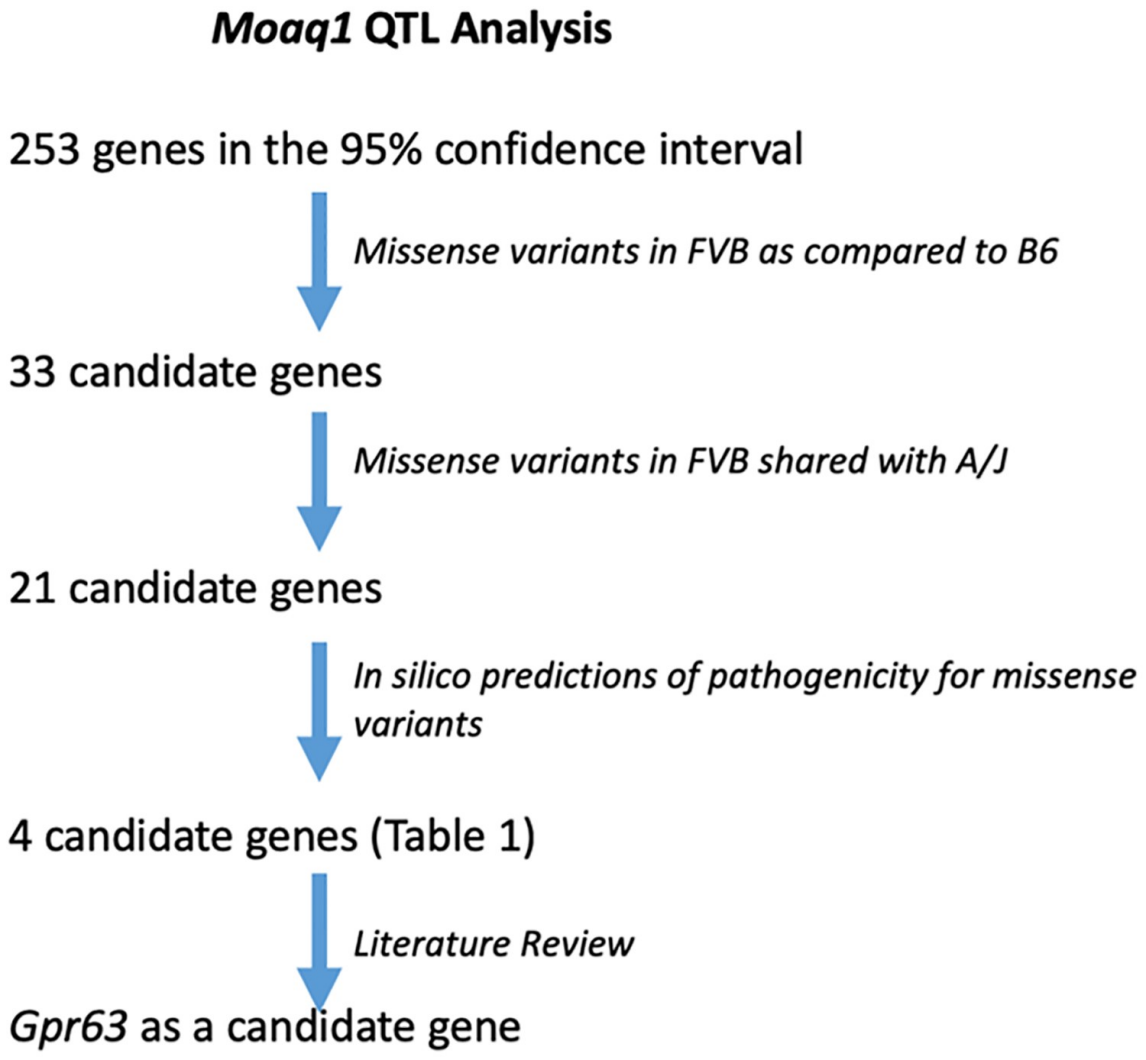
An outline of the analysis leading to the hypothesis *Gpr63* is a causal gene in the *Moaq1* interval.

**Fig 5:**
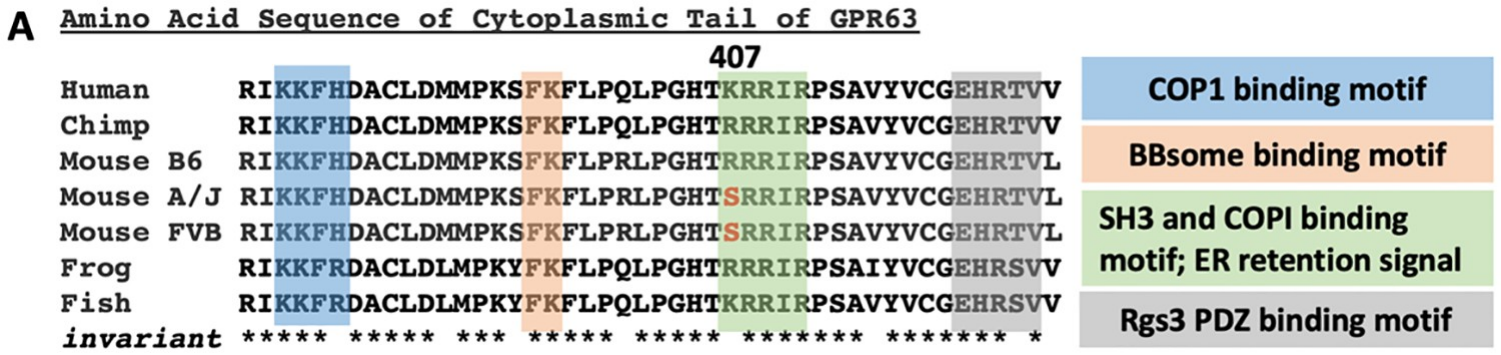
*Gpr63* is a conserved gene. Amino acid sequence of GPR63 from multiple species and three inbred strains of mice. Invariant amino acids are indicated with *. Shading indicates amino acids composing four putative functional domains in the cytoplasmic tail of GPR63.

**Fig 6:**
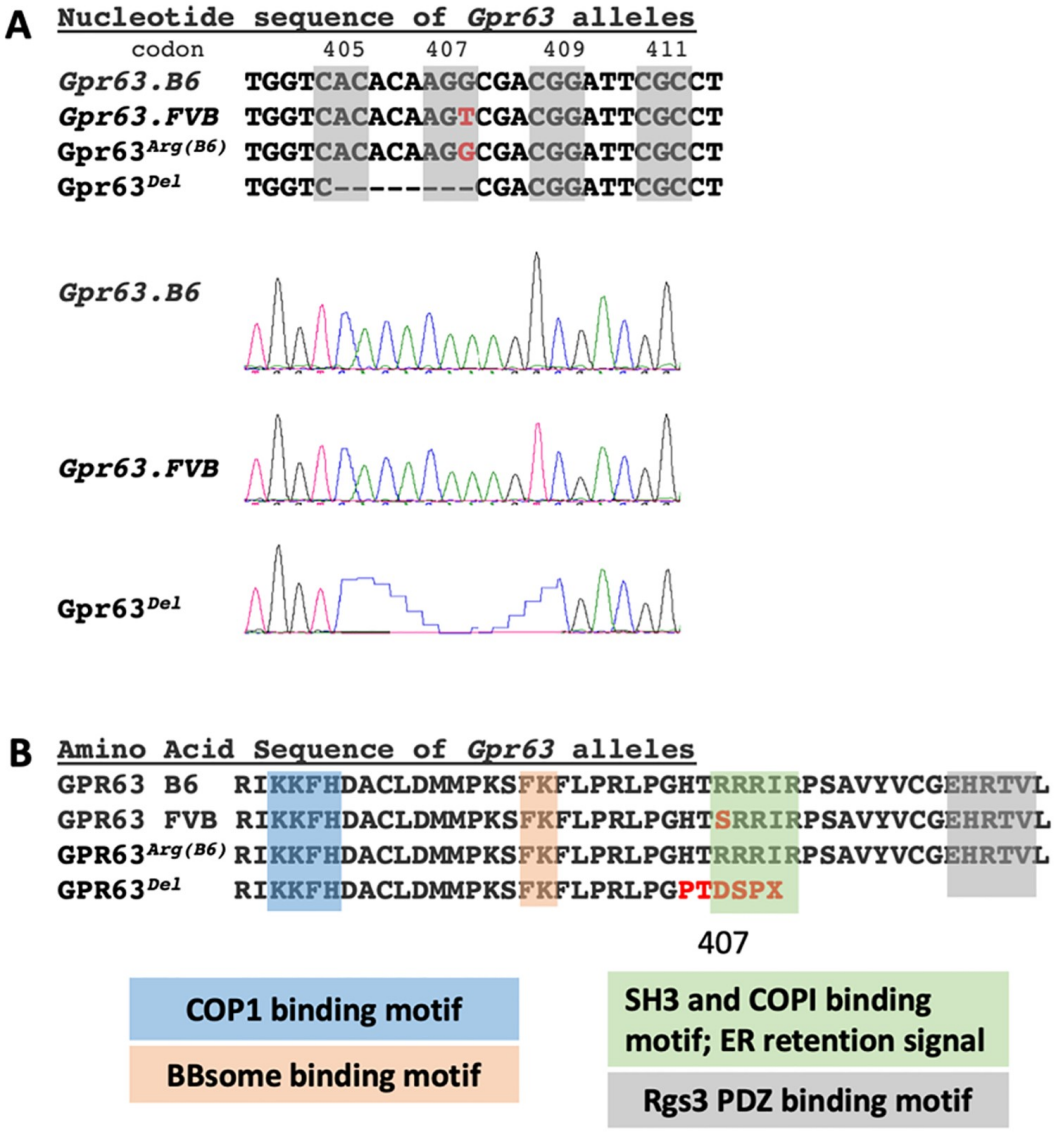
CRISPR-Cas9 alleles of *Gpr63*. (A) Nucleotide sequence and Sanger sequencing of *Gpr63* in the B6/reference strain and FVB strain with the c.G1635T SNP. *Gpr63*^*Arg(B6)*^ is the FVB mouse line with the B6 Thymidine at *Gpr63* c.1635. *Gpr63*^*Del*^ is an 8-bp deletion in the same region of *Gpr63*. (B) Amino acid sequence of the above lines showing the R407S polymorphism between B6 (Arg) and FVB (Ser) reverted to B6 (Arg) in the CRISPR transgenic, and the missense mutation and premature truncation in the *Gpr63*^*Del*^ allele.

In order to identify potential candidate causal genes within the ~26.5 Mb *Moaq1* QTL, the 253 genes within the 95% confidence interval were analyzed (Fig 4; S3A Table). We initially focused our analysis on strain-specific missense coding variants. We recognize that this initial analysis of missense variants, rather than a parallel approach first examining the strain-specific insertions and/or deletions, introduces some ascertainment bias towards different candidate genes. We also could include RNA expression analysis of mutants from both background and look for non-coding regulatory variants within the QTL. However, we initially reasoned that missense coding variants might be best suited to act as modifiers than more severe protein perturbations resulting from insertion/deletion events. We first identified 33 genes with missense polymorphisms when comparing B6 and FVB genomic sequences (S3B Table). The list was further refined to 21 (S3C Table) by selecting only those genes in which the A/J and FVB SNP were identical, given the phenotypic similarities in *Ttc21b*^*aln/aln*^ mutants on those genetic backgrounds. The remaining missense mutations were analyzed with the SIFT prediction algorithm to assess pathogenicity [29], as well as information on known phenotypes in mouse models or human disease (OMIM), expression data and other information from the literature (S3C Table). Four genes were found with predicted deleterious missense mutations (<0.05 SIFT value). We then performed a literature review of these four remaining genes (Table 3, S3D Table) to identify those previously known to have a role in developmental neurobiology and narrowed the list to 3 candidates: *gamma-glutamyl hydrolase (Ggh*, MGI 1329035*)*, *G-protein coupled receptor 63 (Gpr63*, MGI 2135884*)*, and *origin recognition complex subunit 3 (Orc3*, MGI 1354944*)*. *Ggh* is a lysosomal enzyme with a role in folate metabolism, which is provocative given the exencephaly noted in *Ttc21b*^*aln/aln*^ mutants [30]. Furthermore, *Ggh* is the only gene in the interval with 2 potentially deleterious missense mutations. *Orc3* is a nuclear localized component of the Origin Recognition Complex [31, 32]. While conditional ablation of *Orc3* in the brain revealed a requirement in proper neural progenitor and radial glial development, germline loss of *Orc3* led to early embryonic lethality [33]. We next considered any evidence for these remaining candidate genes within the *Moaq1* QTL to have a role in primary cilia biology. Unlike *Ggh* or *Orc3*, *Gpr63* is a membrane protein, making it the more attractive candidate to be a modifier of a mutation in a plasma membrane-based organelle, the primary cilium. An siRNA screen to identify novel regulators of ciliogenesis *in vitro* did provide some evidence *Gpr63* may have a role in cilia biology [34]. *Gpr63* has been reported to have brain-specific expression and to be enriched in the forebrain [35, 36]. The predicted deleterious polymorphic SNP within *Gpr63* (rs13477613; NM_030733 c.G1635T; NP_109658 p.R407S; SIFT score of 0.01) is a missense variant altering the coding of amino acid 407 from Arginine, an amino acid with an electrically charged side chain, in the B6 reference genome to Serine, an amino acid with a polar uncharged side chain, in the A/J and FVB genomes (Table 3). This Arginine residue is conserved from frogs to chimpanzees (Fig 5A), although humans have a Lysine at the orthologous residue in GPR63, an amino acid with an electrically charged side chain like Arginine. The loss of Arginine from the mouse GPR63 cytoplasmic tail alters a predicted RxR endoplasmic reticulum (ER) retention signal (Fig 5) [37]. The C-terminus of GPCRs is understood to play critical roles in protein-protein interactions and regulation of the receptor [38]. As shown in Fig 6A, the c.G1635T SNP changes the amino acid sequence of the coat protein complex I (COPI) binding sequence. COPI is a coatomer responsible for the retrograde transport of vesicles from the *trans*-Golgi network to the *cis*-Golgi and from the *cis*-Golgi to the ER [39, 40]. COPI has been implicated in the retrieval of proteins with exposed RxR motifs [41, 42]. These motifs function to retain proteins in the ER until the RxR containing protein is completely processed and ready to be transported out through the Golgi [37]. Often these proteins escape the RxR retrieval mechanism by masking the motif through interaction with another protein, a critical step in preparing the protein for its function which may include assembling a hetero-multimeric complex [37]. There are candidate motifs for protein domain interactions on the GPR63 C-tail which could serve in this masking role including: LPRLPGH, a predicted SH3 binding motif of DLG4, and EHRTV, a predicted PDZ binding domain of RGS3 [43–46]. We also note a BBSome binding motif which may serve as a potential ciliary targeting sequence [47].

**Table 3:** Candidate *Moaq1* variants.

Gene	Location (Mb)	dbSNP	TRANSCRIPT	NUCLEOTIDE CHANGE	AMINO ACID CHANGE	Mutation Prediction (SIFT)	Other Information
*Ggh*	19,981,227	rs46439394	NM_010281.2	A496G	D125G	Deleterious	Involved in folate metabolism
*Ggh*	19,992,949	rs16796754	NM_010281.2	T1017G	F299V	Deleterious	
*Gpr63*	24,935,645	rs13477613	NM_030733.3	G1635T	R407S	Deleterious	Adult forebrain expression
*Orc3*	34,544,654	rs27788317	NM_01159563.1	G667C	A212P	Deleterious	Null allele early embryonic lethal
*Zfp292*	34,755,239	rs13477642	NM_013889.2	T5097C	C1690R	Low Confidence Deleterious	Possible embryonic forebrain expression

### CRISPR-Cas9 genome editing of *Gpr63*

A standard approach to reduce the number of candidate genes within a QTL is to narrow the genetic interval carrying the trait of interest using meiotic recombination. For this, QTL-interval specific recombinants are identified and overlapping sub-congenics produced and tested to determine the minimal region of effect. This process can be repeated as needed to critically narrow the candidate interval. We initially pursued this strategy, but the results above show that the combination of exencephaly and early lethality of congenic FVB.Cg-*Ttc21b*^*aln/aln*^ mice will preclude successful implementation unless we could also identify the region contributing to the exencephaly.

An alternative approach is to directly test hypotheses about candidate sequence variant(s) with genome editing to create novel transgenic models. We chose to pursue this and targeted FVB mice to change the rs13477613 polymorphism from FVB sequence (c.1635-T; coding for *Gpr63*^*Ser(FVB)*^) to the reference, non-pathogenic, B6 sequence (c.1635-G; coding for *Gpr63*^*Arg(B6)*^). This allele *Gpr63*^*em1Rstot*^, is hereafter referred to as FVB*-Gpr63*^*Arg(B6)*^. An added benefit of CRISPR-Cas9 genome editing is the potential to recover an allelic series from the initial set of transgenic founders. We recovered and maintained a second allele *Gpr63*^*em2Rstot*^ (*Gpr63*^*Del*^), with an 8-bp deletion resulting in a frameshift mutation that leads to the translation of 5 missense amino acids and an early stop, thus preventing translation of the last 21 amino acids of the GPR63 C-terminus (Fig 6A). This *Gpr63*^*Del*^ mutation may disrupt both an ER retention signal and an RGS3 PDZ binding motif, creating a potentially more deleterious mutation than *Gpr63*^*Ser(FVB)*^ [37, 43–46].

**Fig 7:**
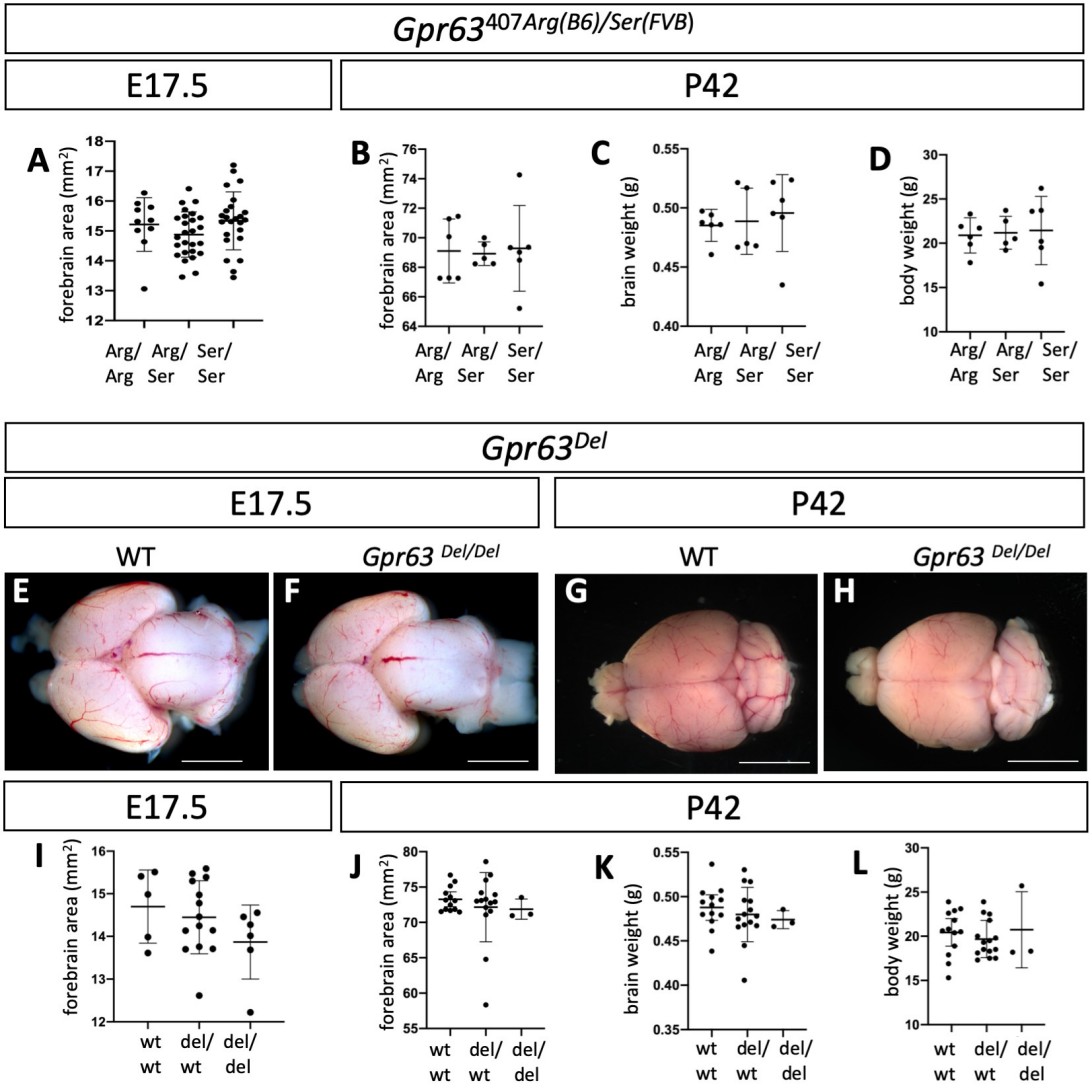
*Gpr63* alleles do not affect survival or overall brain size and morphology. (A-D) Animals with *Gpr63*^*407Arg/Arg*^, *Gpr63*^*407Arg/Ser*^, or *Gpr63*^*407Ser/Ser*^ do not vary in forebrain area at E17.5 (A) or at P42 in forebrain area (B), brain weight (C) or overall body weight (D). (E-L) Animals homozygous for the cytoplasmic deletion of *Gpr63* (*Gpr63*^*Del/Del*^) also have morphologically normal brains at E17.5 (E,F) and P42 (G,H). (I) Forebrain area at E17.5 is unaffected. At P42, forebrain area (J), brain weight (K) and overall body weight (L) are unaffected by the deletion allele. Scale bars indicate 2mm in E,F and 5mm in G,H.

We first determined that the *Gpr63*^Arg/Ser^ polymorphism did not affect survival or brain size at E17.5 (Fig 7A, ANOVA p = 0.1743). We also did not see an effect at postnatal day (P) 42 on either forebrain dorsal surface, brain weight or total body weight (Fig 7B–7D, ANOVA p = 0.9639, 0.7753, 0.9420, respectively). This was expected, given that these are sequence polymorphisms found between two commonly maintained inbred strains. We hypothesized the *Gpr63*^*Del*^ allele may be more deleterious than the *Gpr63*^*Ser(FVB)*^ polymorphism alone. We first determined if this allele affects survival and created animals homozygous for the *Gpr63*^*Del*^ allele. We recovered *Gpr63*^*Del/Del*^ mutants in approximately Mendelian ratios at weaning (8/35) and they survived to adulthood (n = 8) with no discernible phenotypes seen to date. We measured forebrain dorsal surface, brain weight and body weight at both E17.5 and P42 and found no change in the *Gpr63*^*Del/Del*^ mutants (Fig 7E–7L; ANOVA p = 0.2569, 0.6846, 0.6344, 0.6655, respectively). Consistent with the predicted deleterious effects of the SNP at amino acid 407 seen in both viable and fertile FVB and A/J animals, this suggests that *Gpr63* does not have an essential requirement in development.

### *Gpr63* expression *in vitro* shows ciliary localization

**Fig 8:**
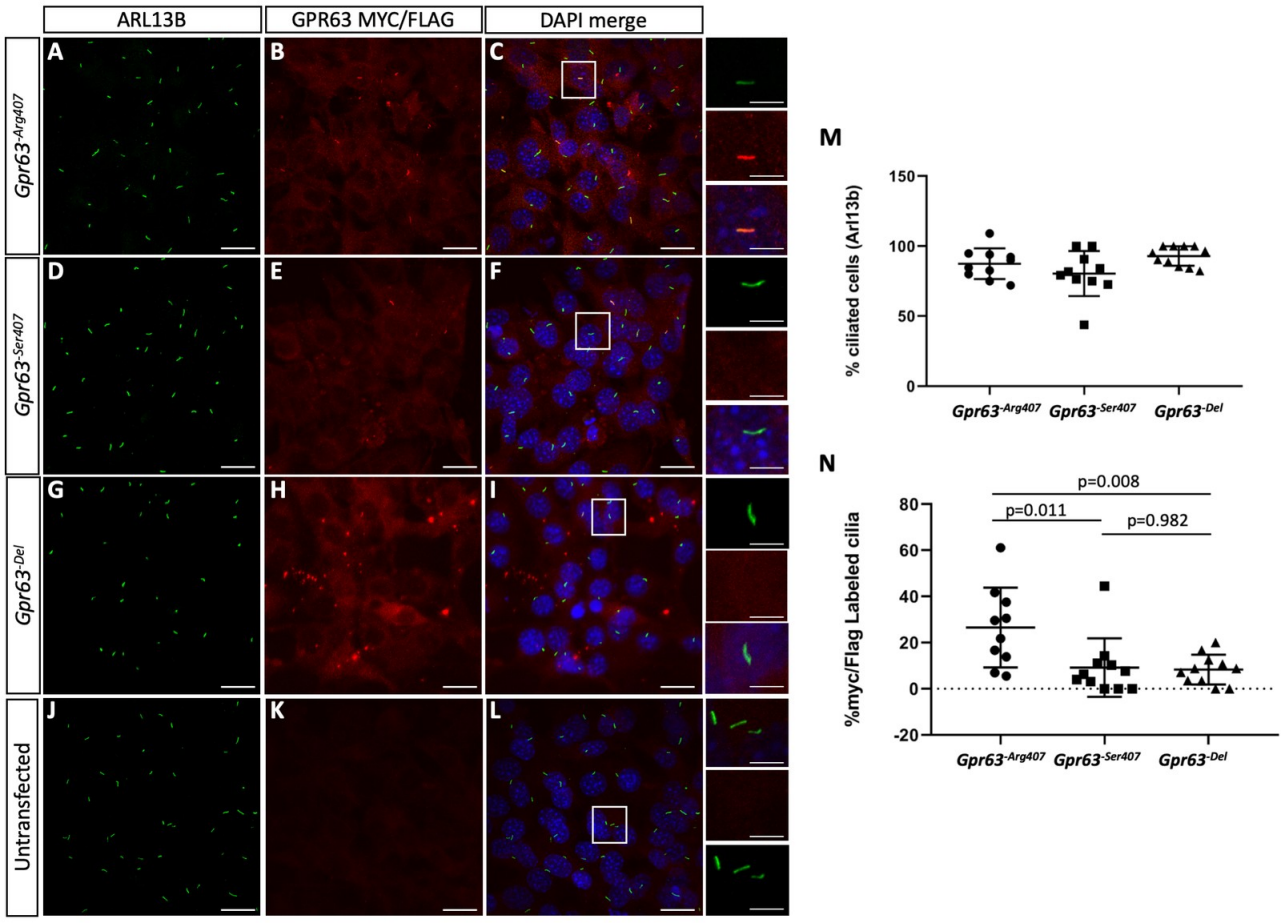
Ciliary localization is altered by overexpression of *Gpr63* variants. NIH 3T3 cells were transfected to overexpress *Gpr63*^*Arg407*^ (A-C), *Gpr63*^*Ser407*^ (D-F) or *Gpr63*^*Del*^ (G-I) constructs. ARL13B was used as a marker of the ciliary axoneme. (J-L) An untransfected immunocytochemistry control is shown. Small panels show higher magnification of cells highlighted by white boxes to show cilia with robust, no, or little GPR63 immunoreactivity. (M) Expression of *Gpr63* did not appear to affect the number of cells producing cilia. (N) *Gpr63*^*Ser407*^ and *Gpr63*^*Del*^ constructs result in reduced ciliary accumulation of the epitope-tagged GPR63. Scale bars represent 20μm in A-L and 5μm in the inset panels.

We directly tested the hypothesis that *Gpr63* might, at least partially, localize to the primary cilium by measuring *in vitro* expression of a myc-flag-epitope tagged construct in NIH 3T3 cells (pCMV6-GPR63-myc). Indeed, we found colocalization of the GPR63 signal with Arl13b as a marker of the ciliary axoneme (Fig 8A–8C). We were not able to identify a completely reliable antibody to verify these findings, but saw similar results from an independent GPR63 expression construct (GPR63-TANGO-FLAG [48], S2 Fig). Site-directed mutagenesis was used to generate constructs recapitulating the variants seen *in vivo* (Fig 8D–8I). Overexpression of either polymorphism of *Gpr63* or the *Gpr63-*deletion construct did not affect the proportion of ciliated cells (Fig 8M, ANOVA p = 0.067). However, when we expressed either the myc-*Gpr63*^*Ser*^ or the myc-*Gpr63*^*Del*^ construct we saw a reduction in the number of transfected cells with GPR63 in the primary cilia (Fig 8N, ANOVA p = 0.004). We saw a 65% decrease with *Gpr63*^*Ser407*^ as compared with *Gpr63*^*Arg407*^ (p = 0.011) and a 68% decrease with the *Gpr63*^*Del*^ construct (p = 0.008). We conclude that the different alleles changing the protein composition of the GPR63 cytoplasmic tail can alter the subcellular (i.e., ciliary) localization of GPR63.

### *Alleles of Gpr63* modify *Ttc21b*^*aln/aln*^ mutant forebrain phenotype

The FVB*-Gpr63*^*Arg(B6)*^ animals were crossed with FVB.Cg-*Ttc21b*^*aln/wt*^ (which will have the *FVB* allele of Gpr63: *Gpr63*^*Ser(FVB)*^*)* mice to produce FVB.Cg-*Ttc21b*^*aln/wt*^;*Gpr63*^*Arg(B6)/Ser(FVB)*^ double heterozygotes. This cross was designed to directly test the hypothesis that the predicted pathogenic polymorphism of *Gpr63 (i*.*e*., Ser407*)* contributes to the more severe microcephaly seen in FVB.Cg-*Ttc21b*^*aln/aln*^ homozygous mutants. However, we were unable to directly test this hypothesis. As this experiment was done concurrently with the backcrosses to produce FVB congenic lines described above, we also found that both FVB.Cg-*Ttc21b*^*aln/aln*^;*FVB*.*Gpr63*^*Arg(B6)/Arg(B6)*^ and FVB.Cg-*Ttc21b*^*aln/aln*^;*FVB*.*Gpr63*^*Ser(FVB)*/*Ser(FVB)*^ mice have exencephaly at a high frequency precluding measurement of forebrain size in these animals.

**Fig 9:**
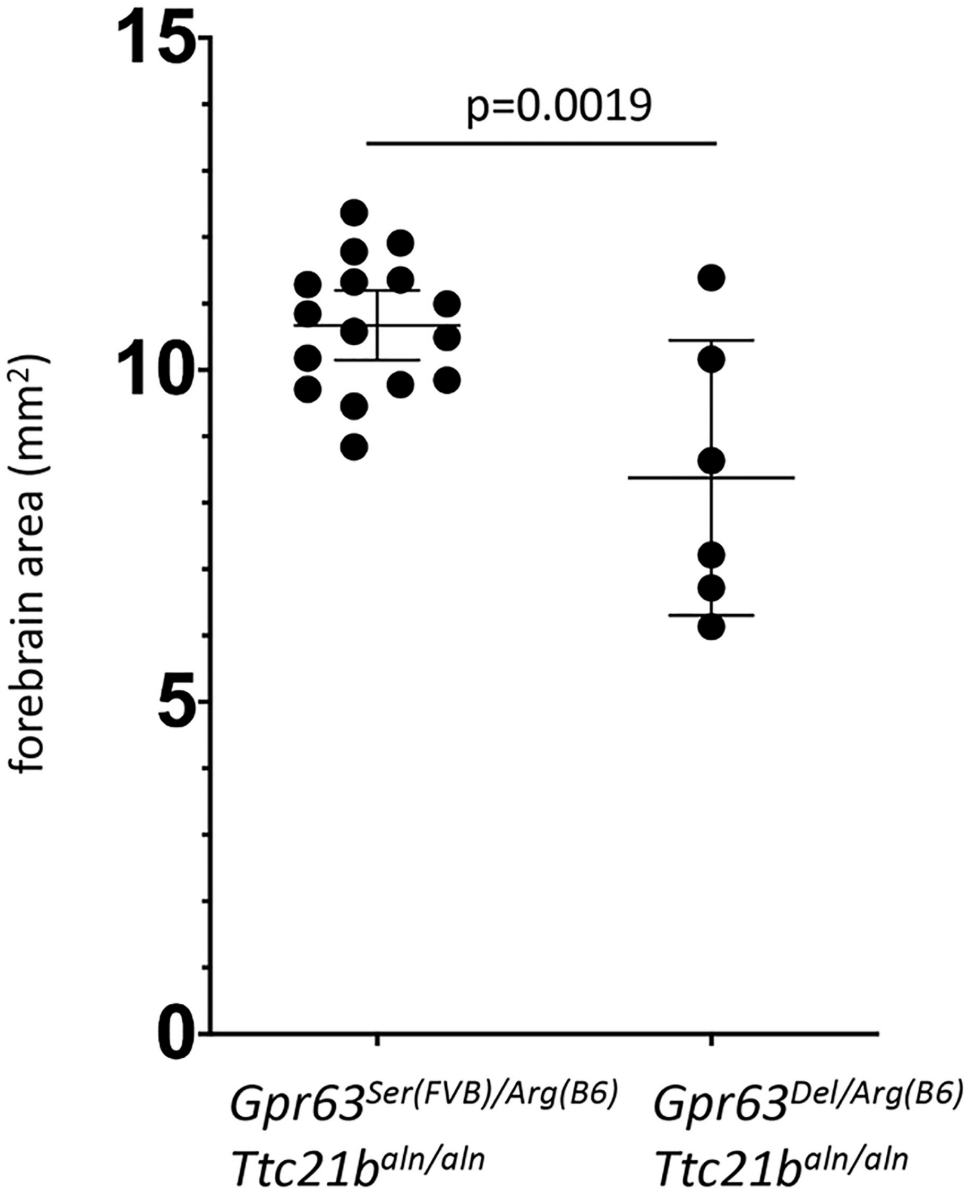
*Gpr63* alleles affect forebrain area in *Ttc21b*^*aln/aln*^ mutants. Forebrain area in FVB;B6-*Ttc21b*^*aln/aln*^;*Gpr63*^*Arg(B6)/Ser(FVB)*^ E17.5 embryos (n = 16) is larger than FVB;B6-*Ttc21b*^*aln/aln*^-*Gpr63*^*Del/Arg(B6)*^ animals (n = 6), p = 0.0019.

In order to test for a genetic interaction between *Gpr63* and *Ttc21b*, we created FVB.Cg-*Ttc21b*^*aln/wt*^-*Gpr63*^*Del/Ser(FVB)*^ mice and crossed these with B6.Cg-*Ttc21b*^*aln/wt*^ (with no modification of the *Gpr63* locus, e.g., B6.Cg-*Ttc21b*^*aln/wt*^-*Gpr63*^*Arg(B6)/Arg(B6*^). We hypothesized that reduced GPR63 function in the resulting F1 FVB;B6-*Ttc21b*^*aln/aln*^;*Gpr63*^*Del/Arg(B6)*^ embryos as compared to F1 FVB;B6-*Ttc21b*^*aln/aln*^*-Gpr63* ^*Ser(FVB)/Arg(B6)*^ will lead to a smaller forebrain in the F1 FVB;B6-*Ttc21b*^*aln/aln*^*-Gpr63*^*Del/Arg(B6)*^ animals (i.e., deletion of the GPR63 cytoplasmic tail is more deleterious than a single Ser/Arg amino acid change). Indeed, F1 FVB;B6-*Ttc21b*^*aln/aln*^*-Gpr63*^*Del/Arg(B6)*^ mice had an approximate 25% decrease in forebrain area (Fig 9; -2.30 mm^2^; n = 15 and n = 6; p = 0.0019). This experiment demonstrates that alleles of *Gpr63* can modify the *Ttc21b*^*aln/aln*^ forebrain phenotype and supports the conclusion that *Gpr63* genetically interacts with *Ttc21b*.

To further address a possible interaction between *Ttc21b* and *Gpr63*, we aimed to create *Ttc21b*^*aln/aln*^;*Gpr63*^*Del/Del*^ double homozygous mutant animals. Note this experiment was done in a way that necessitated crosses with differing mixtures of the FVB and B6 background. Therefore, the *Gpr63;Ttc21b* double mutant analysis is best considered on a hybrid background with *Gpr63* alleles being the only relevant variable. We recovered 8 *Ttc21b*^*aln/aln*^;*Gpr63*^*Del/Del*^ embryos at E17.5 and E18.5 from this cross and found that 75% (6/8) were embryonic lethal and had died around E12.5. The remaining two had exencephaly precluding measurement of forebrain area.

**Fig 10:**
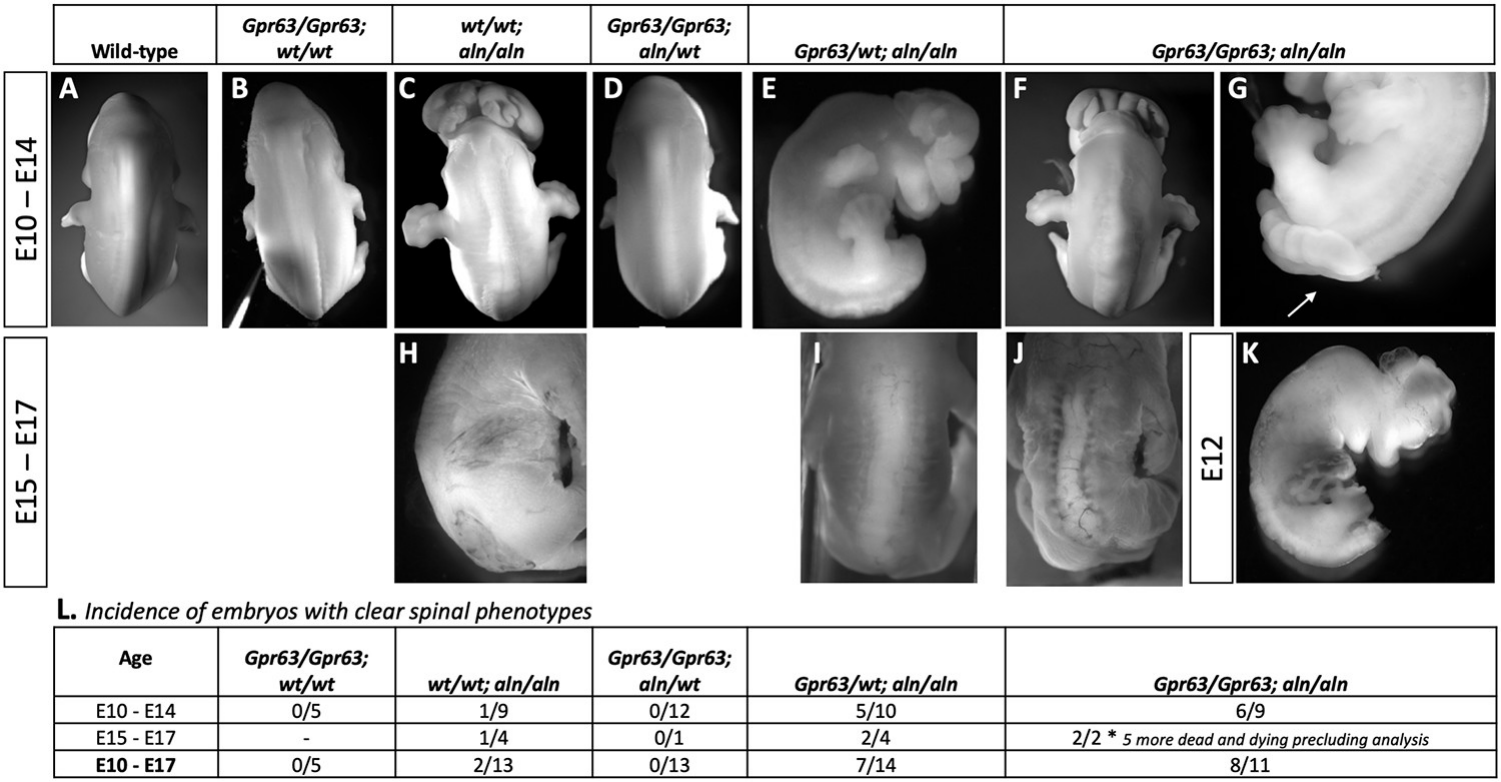
*Gpr63*^*del/del*^;*Ttc21b*^*aln/aln*^ double mutants have increased incidence of neural tube defects. Embryos of multiple genotypes from a *Gpr63*^*del/wt*^;*Ttc21b*^*aln/wt*^ intercross are shown at stages from E10-14 (A-G, K) or E15-17 (H-J). *Gpr63*^*del/del*^;*Ttc21b*^*aln/aln*^ double mutants do not survive to term and show multiple phenotypes which are more severe than either single mutation including spina bifida aperta (G). (L) Incidence of neural tube closure defects of any severity in the embryos resulting from the *Gpr63*^*del/wt*^;*Ttc21b*^*aln/wt*^ intercross. Note the dramatic increase in penetrance in double mutants (8/11) as compared to any other genotype.

We further explored this phenotype at ~E12.5 (E10.5–14.5) and recovered 9 *Ttc21b*^*aln/aln*^*-Gpr63*^*Del/Del*^ double homozygous null embryos (Fig 10F). We saw many of the phenotypes previously noted in our studies of *Ttc21b*^*aln/aln*^ mutants. These included 89% (8/9) with exencephaly, and 100% with polydactyly in both the forelimb and hindlimb (9/9). Surprisingly, we also noted phenotypes never seen in any *Ttc21b*^*aln/aln*^ mutant. One of the double mutant embryos had quite dramatic spina bifida aperta (Fig 10G), a severe neural tube defect. 62.5% (5/8) of the remaining embryos had morphology consistent with the considerably milder spina bifida occulta. Both of the previously mentioned ~E17.5 double mutant embryos also had obvious spina bifida occulta (Fig 10J). We then rigorously examined all the embryos recovered from this cross for spinal neural tube defects (NTD) as this is not a phenotype we had seen in previous analyses of the *Ttc21b*^*aln/aln*^ mutants [16, 19]. We did not note any spinal NTDs in *Gpr63*^*Del/Del*;^ *Ttc21b*^*wt/wt*^ (Fig 10B; n = 5) or *Gpr63*^*Del/Del*;^ *Ttc21b*^*aln/wt*^ embryos (Fig 10D; n = 13). In *Gpr63*^*wt/wt*;^ *Ttc21b*^*aln/aln*^ embryos, we did note a previously unappreciated thickened neural tissue in one mutant from E10-E14 (Fig 10C; n = 1/9), and a disruption of the overlying ectoderm possibly consistent with spina bifida occulta from E15-17 (Fig 10H; n = 1/4). Based on our previous studies with the *Ttc21b*^*aln/aln*^ mutant where the NTDs were not noticed in studies performed on similar genetic backgrounds, we believe these to be relatively uncommon phenotypes in mice with just the *Ttc21b*^*aln*^ allele [16, 19]. *Gpr63*^*Del/wt*;^ *Ttc21b*^*aln/aln*^ embryos with an additional deleterious *Gpr63* allele however, showed a significantly increased incidence of NTDs (Fig 10E and 10I, n = 7/14). However, we never see the spina bifida aperta phenotype in embryos other than *Gpr63*^*Del/Del*;^ *Ttc21b*^*aln/aln*^ double mutants. Even in this genotype, we have seen it once in this study. We therefore suggest it is a relatively rare phenotype, but is still restricted to the *Gpr63*^*Del/Del*;^ *Ttc21b*^*aln/aln*^ double mutant genotype.

Thus, we conclude that an allele of *Gpr63* with a truncation of the cytoplasmic tail genetically interacts with *Ttc21b*, such that double mutants are mostly embryonic lethal at stages prior to any lethality seen in either single mutant. The remaining animals have an increased incidence of NTDs unique from either single mutant. The mechanism(s) leading to this lethality will be the subject of future study. We further suggest that *Gpr63* is likely to be a causal gene within the *Moaq1* QTL and reduced function of GPR63 in the FVB and A/J genetic backgrounds leads to the more severe forebrain phenotype of the *Ttc21b*^*aln/aln*^ homozygous mutants on these backgrounds, as compared to those recovered on a B6 inbred background.

## Discussion

In this study, we sought to understand the genetic basis for the influence of mouse inbred strain background on the severity of the *Ttc21b*^*aln/aln*^ homozygous mutant microcephaly phenotype. Our QTL analysis identified two loci (*Moaq1*, *Moaq2)* which interact with *Ttc21b* to control brain size in *Ttc21b*^*aln/aln*^ mutants. These were validated by constructing QTL-containing congenic lines for each. The 95% confidence interval of *Moaq1* was small enough to allow a candidate gene approach, and *Gpr63* was selected for further study. Experiments directly testing *Gpr63* function with novel mouse alleles generated with CRISPR-Cas9 genome editing support our hypothesis that reduced *Gpr63* function further compromises embryos lacking functional *Ttc21b*. We further demonstrated that *Gpr63;Ttc21b* double mutants had earlier lethality than either single mutant as well as novel spina bifida phenotypes.

### Meiotic recombination or genome editing

Traditionally, the identification of a QTL is followed by a laborious mapping process and the creation of congenic strains to validate the QTL and begin to hunt for the truly causal sequence difference [49, 50]. The combination of deeply sequenced inbred mouse strains, combined with the capabilities conferred by new genome editing tools such as CRISPR-Cas9 to rapidly generate sequence specific alterations in novel mouse lines, can potentially greatly facilitate the identification of causal genetic variants within a QTL. In this case, we identified a potentially deleterious SNP in *Gpr63* on the FVB and A/J genetic backgrounds which may cause reduced function as compared to the canonical sequence seen in B6 mice. We did pursue a traditional congenic backcross approach by continued backcrossing to FVB towards creating a true congenic line which would be “pure” FVB with only dilution by the *Ttc21b*^*aln*^ locus itself and the B6 *Moaq1* region. However, this was confounded by the increased incidence of exencephaly and/or early embryonic lethality seen in *Ttc21b*^*aln/aln*^ mutants when maintained on the >90% FVB genetic background. Presumably this increased frequency in exencephaly and/or death was due to the remaining 10% of the heterozygous genome that was further purified to FVB during this backcrossing. This is an intriguing finding that could potentially facilitate mapping those modifiers independently. In parallel, we attempted to bypass the meiotic recombination and QTL refinement entirely by recreating the specific *Gpr63* SNP and determining if it affected brain size in the *Ttc21b* mutant background. In the F2 recombinants generated for the QTL analysis, we did not recover embryos both without exencephaly and homozygosity for B6 at a region on chromosome 14. This suggested to us that the *Ttc21b*^*aln*^ allele on a pure B6 background may be early embryonic lethal; therefore, we chose to test our *Gpr63* hypothesis by creating the B6 SNP on an FVB background (FVB *Gpr63*^*Arg(B6)/Ser(FVB)*^). Further study has shown this lack of homozygosity on chromosome 14 was a statistical anomaly with no apparent biological significance as we have since recovered *Ttc21b*^*aln/aln*^ mutants with B6 identity across this region. In contrast, the consequences of creating a pure inbred FVB strain with the *Ttc21b* allele was a strain with a high incidence of exencephaly preventing us from testing this *Gpr63* hypothesis directly. We are now carrying out a further validation of the *Moaq1* and *Moaq2* QTL by constructing pure B6 congenics with FVB *Moaq1* and *Moaq2* intervals.

### *Gpr63* can localize to the primary cilia but is not essential for development

Our data suggest that the predicted deleterious *Gpr63*^*407Arg(B6)/Ser(FVB)*^ SNP is not essential for development. This is consistent with the fact this SNP is present in the reference sequence for A/J and FVB mice. Furthermore, the deletion of the extreme C-terminus of the GPR63 GPCR also did not seem to have an effect on survival (Fig 7). This is consistent with data from the International Mouse Phenotyping Consortium and Knockout Mouse Project that *Gpr63* deletion alleles do not affect survival (https://www.mousephenotype.org/data/genes/MGI:2135884).

We did use two different epitope-tagged expression constructs to demonstrate GPR63 at least partially localizes to the primary cilium (Fig 8). This is consistent with the BBSome binding motif located in the C-terminus. We further studied the ciliary localization of different alleles of *Gpr63* in these overexpression experiments and have provided evidence that the Arg/Ser transition and the cytoplasmic truncation reduced ciliary localization of GPR63 (Fig 8). Neither of these residues affect the presumed BBSome binding motif, suggesting that other residues contribute to the ciliary targeting of GPR63 through currently unknown mechanisms. The detailed analysis of this trafficking motif and potential physical interaction with TTC21B should be the subject of future study.

### *Gpr63* and *Ttc21b* genetic interactions

The alleles we generated with CRISPR-Cas9 editing did allow us to demonstrate a genetic interaction between *Gpr63* and *Ttc21b*. The confounding effects of FVB genetic purity on exencephaly and the apparent chromosome 14 lethality interval did hamper some of the initially designed experiments. However, the truncated *Gpr63* allele we recovered from our CRISPR-Cas9 studies allowed us to address this hypothesis and we do see that *Ttc21b*^*aln/aln*^;*Gpr63*^*Del/Arg(B6)*^ embryos have smaller forebrain tissues when compared to *Ttc21b*^*aln/aln*^;*Gpr63* ^*Ser(FVB)/Arg(B6*^ (Fig 9). The best evidence for a genetic interaction between *Gpr63* and *Ttc21b*, however, is the novel phenotypes seen in the *Ttc21b*^*aln/aln*^;*Gpr63*^*Del/Del*^ embryos. The lethality of these embryos is earlier than what we observed in *Ttc21b*^*aln/aln*^ mutants and we have never previously seen some of the spina bifida phenotypes observed in the *Ttc21b*^*aln/aln*^;*Gpr63*^*Del/Del*^ embryos.

Here we identify regions of the genome linked to the differential effect of inbred strain background on the severity of the microcephaly phenotype seen in *Ttc21b*^*aln/aln*^ mutants. We follow this up with a new allelic series of *Gpr63* and experiments *in vitro* and *in vivo* to demonstrate these polymorphisms do affect *Ttc21b* phenotypes and *Gpr63* function. Although loss of *Gpr63* alone does not seem to cause a significant phenotype, this work suggests *Gpr63* may be a risk factor underlying some of the variability seen in the ciliopathies. The approach taken here may be quite useful in understanding modifier effects in any number of structural birth defects.

## Materials and methods

### Ethics statement

All animals were housed under a protocol approved by the CCHMC Institutional Animal Care and Use Committee (#2016–0098) in standard conditions. All euthanasia and subsequent embryo or organ harvests were preceded by Isoflurane sedation. Euthanasia was accomplished via dislocation of the cervical vertebrae.

### Animal husbandry

For embryo collections, noon of the day of vaginal plug detection was designated as E0.5. The *Ttc21b*^*aln*^ allele used in this study has been previously published [16]. *Ttc21b*^*aln*^ mice were serially backcrossed to C57BL/6J (JAX:000664) and FVB/NJ (JAX:001800) mice to produce strain specific *Ttc21b*^*aln*^ mice. Genotyping was performed by PCR, Sanger Sequencing, or Taqman assays (S4 Table).

### Forebrain surface area measurement

Brains were microdissected from embryos and photographed prior to fixation from the dorsal aspect on a Zeiss Discovery.V8 dissecting microscope. Zeiss Axiovision software was used to mark the boundaries of the cortical surface area for all brain examined, as shown in S1 Fig. Cortical surface area was quantified by the Zeiss Axiovision software and recorded. All images used to quantify surface area for the QTL analysis were taken on the same microscope at the same magnification.

### QTL analysis

GigaMUGA and MegaMUGA genotyping of recombinants was performed by GeneSeek (Neogen; Lincoln, NE). Quality and intensity normalization of the SNP data was checked using routines provided in the R-package Argyle [51]. Linkage mapping, including scanone and scantwo analyses, was performed on informative SNP markers and brain sizes (mm^2^) of ninety-six F_2_ recombinants using the R/qtl software package (Broman 2003; Broman and Sen 2009). Threshold levels of significance were established using at least 1,000 permutations of the respective dataset.

Coding differences of candidate genes were compared using the Mouse Genomes Project website from Wellcome Sanger Trust Institute (https://www.sanger.ac.uk/sanger/Mouse_SnpViewer/rel-1211, NCBIm37).

### CRISPR-Cas9 genome editing

Donor oligonucleotide and guide RNA vector sequences were designed using Benchling (Benchling, 2016). sgRNAs were validated using Benchling and CRISPRscan with the following scores: Benchling-48.5 and CRISPRscan-49 (Benchling, 2016) (Moreno-Mateos, et al., 2015). The donor oligonucleotide was supplied by Integrated DNA Technologies (sequence with lower case letters representing coding change: CCATAATCGCTCGATATGTTTCAAGAGTTCGGTATTCACAACACCGTCCGATGTTCCCCACACACGTAGACGGCACTAGGGCGAATgCGTCGcCTTGTaTGcCCgGGGAGCCGTGGCAAGAACT. The sgRNA (sequence: TCCCTGGTCACACAAGTCGA) was synthesized and injected into FVB mouse zygotes together with donor oligonucleotide by the CCHMC Transgenic Animal and Genome Editing Core. *Gpr63* mice were made by injection into FVB zygotes. The male founder mice were genotyped and those with evidence of edited GPR63 loci were bred to FVB-*Ttc21b*^*aln/wt*^ females. Offspring were analyzed by PCR and Sanger sequencing of approximately 1000 base pairs of the *Gpr63* locus around the sgRNA recognition sequence. Initial crosses were designed to rapidly assess the hypothesized interaction between *Gpr63* and *Ttc21b*. Carriers for the SNP allele (*Gpr63*^*Arg(B6)*^) were maintained by further breeding to FVB-*Ttc21b*^*aln/wt*^ females to immediately ascertain an effect on brain size. These were then further maintained on FVB to characterize the *Gpr63* phenotype in isolation. Carriers for the *Gpr63*^*Del*^ allele were also bred to *FVB-Ttc21b*^*aln/wt*^ (for at least two generations). In order to compare *Gpr63*^*Arg*^ with the *Gpr63*^*Del*^ allele, *Gpr63*^*Del*^;*FVB-Ttc21b*^*aln/wt*^ mice heterozygous for both alleles were crossed to B6 wild-type mice.

### Cell culture and transfection

A pCMV6-GPR63-myc (Origene, Rockville, MD) or GPR63-TANGO-FLAG [48] (Addgene.org) expression plasmid was transfected into NIH 3T3 cells at ~80% confluency using Lipofectamine 3000 and incubated for 2–3 days. The pCMV6-GPR63-myc plasmid has a myc-DDK tag at the C-terminus of human *GPR63* driven by a CMV promoter in the pCMV6 expression vector. GPR63-TANGO-FLAG is a plasmid designed to express human *GPR63* with an N-terminal FLAG tag under the control of a CMV promoter from a Tango vector [48]. Mutations were made to the pCMV6-GPR63-myc expression plasmid to mimic the *Gpr63*^*Ser*^ and *Gpr63*^*Del*^ mouse alleles (Genscript, Piscataway, NJ). To induce ciliogenesis, cells were grown to confluency and serum starved. Immunocytochemistry was performed with a mouse anti-myc (Sigma M4439; 1:500), mouse anti-FLAG (Sigma F3165; 1:500), rabbit anti-Arl13b (Proteintech17711-1-AP; 1:500), and anti-mouse acetylated-tubulin (SIGMA T6793; 1:2000) antibodies to detect cilia and GPR63 localization. Secondary antibodies were Alexa Fluor-488, Alexa Fluor-594 Goat anti-mouse/rabbit (Invitrogen, A11001, A20980; 1:500). Confocal imaging was performed on a Nikon C2 system and analysis was performed with Nikon Elements software.

Analysis of localization of GPR63-myc was done with Imaris 6.2.1 software. First, a surface overlying cilia axonemes labeled with Arl13b was initiated in the 488 channel, then another surface was initiated in the 596 channel to detect the cilia labeled with myc and flag. Finally, the MATLAB plugin surface-surface colocalization was utilized to detect the areas of colocalization between Arl13b stained cilia and myc-flag tagged cilia axoneme. The number of double labeled cilia were normalized against the total number of Arl13b stained cilia per field of view. Statistical analysis and One-way ANOVA was performed using GraphPad Prism software. All samples were blinded to the investigator performing the analysis.

### GPR63 protein domain analysis

Features of the GPR63 protein were identified through literature searches and the Modular Domain Peptide Interaction Server: http://modpepint.informatik.uni-freiburg.de/.

### Statistical analysis

GraphPad Prism was used for all graphical analysis. Data are shown as mean ± 95% confidence interval unless otherwise noted. P values are explicitly stated in almost every instance, rather than asserting significance at a certain conventional threshold (e.g., p = 0.05). Fig 1G: Two-way ANOVA followed by unpaired t-test of each strain wild-type vs mutant; Figs 2D, 2F, 7A–7D, 7I–7L and 7O: One-way ANOVA followed by Tukey’s multiple comparisons when ANOVA indicates significance; Figs 2E, 2G and 3 –paired student t-test; Fig 9 –unpaired students t-test.

## Supporting information

S1 Fig Area measured to determine brain size. Shaded area in B indicates area from brain in A measured as “forebrain area”.
(TIF)

S2 Fig GPR63-TANGO-FLAG expression. Transfection of a second GPR63-TANGO-FLAG expression plasmid [48] indicates ciliary localization similarly to the results with pCMV6-GPR63-myc.
(TIF)

S1 Table Genome-wide SNP data. Genome-wide SNP data to determine background purity; used for initial congenic FVB and B6 strains carrying *Ttc21b*^*aln*^ allele.
(XLSX)

S2 Table Genome survey and brain size of F2 mutants. GigaMuga data and brain size of 96 F2 mutants used for initial QTL analysis.
(XLSX)

S3 Table Analysis of candidate genes within *Moaq1* QTL. (XLSX)

S4 Table Genotyping primers used in this study. (XLSX)
