# Supplementary figures and images for: Gpr63 is a modifier of microcephaly in Ttc21b mouse mutants
Source: PLoS Genet. 2019 Nov 15;15(11):e1008467. doi: 10.1371/journal.pgen.1008467 (PMC6881074; doi:10.1371/journal.pgen.1008467)

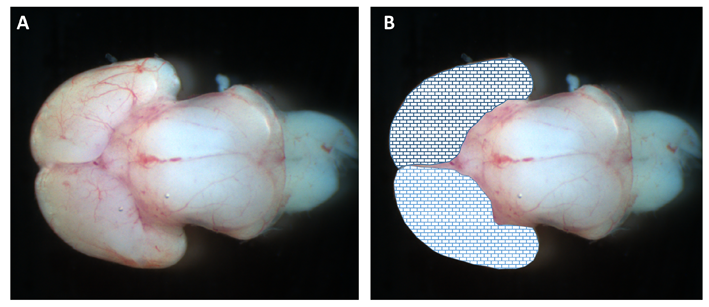

Supplement: S1 Fig — Shaded area in B indicates area from brain in A measured as “forebrain area”. (TIF) [file pgen.1008467.s001.tif]

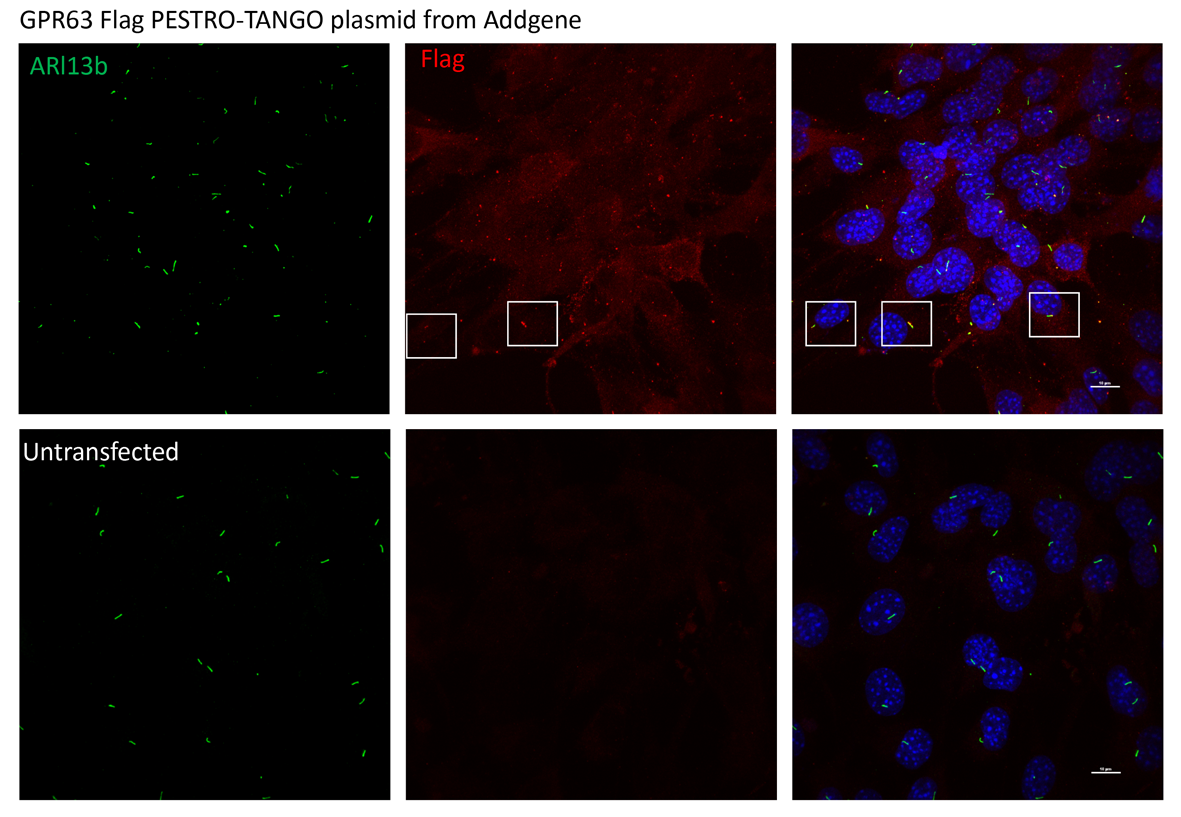

Supplement: S2 Fig — Transfection of a second GPR63-TANGO-FLAG expression plasmid [48] indicates ciliary localization similarly to the results with pCMV6-GPR63-myc. (TIF) [file pgen.1008467.s002.tif]
